# Comparing major and mild cognitive impairment risks in older type-2 diabetic patients: a Danish register-based study on dipeptidyl peptidase-4 inhibitors vs. glucagon-like peptide-1 analogues

**DOI:** 10.1007/s00415-024-12300-9

**Published:** 2024-03-22

**Authors:** Vera Battini, Maria Antonietta Barbieri, Carla Carnovale, Edoardo Spina, Emilio Clementi, Maurizio Sessa

**Affiliations:** 1 Department of Drug Design and Pharmacology, University of Copenhagen, Jagtvej 160, 2100 Copenhagen, Denmark; 2 Pharmacovigilance and Clinical Research, International Centre for Pesticides and Health Risk Prevention, Department of Biomedical and Clinical Sciences (DIBIC), ASST Fatebenefratelli-Sacco University Hospital, Università Degli Studi Di Milano, Milan, Italy; 3 Department of Clinical and Experimental Medicine, University of Messina, 98125 Messina, Italy; 4 Scientific Institute, IRCCS E. Medea, Bosisio Parini, LC Italy

**Keywords:** Glucagon-like peptide-1 analogues, Dipeptidyl peptidase-4 inhibitors, Major cognitive impairment, Mild cognitive impairment, Older individuals, Register-based study

## Abstract

**Introduction:**

The prevalence of major and mild cognitive impairment (CI) in type-2 diabetes older patients is 15–25% and 30–60%, respectively, thus affecting quality of life and health outcomes. There is, therefore, the need of head-to-head studies aiming at identifying the optimal treatment for individuals with type-2 diabetes at increased risk of mild and major CI. This study focuses on the risk of developing mild and major CI in Danish patients treated with dipeptidyl peptidase-4 inhibitors (DPP-4i) and glucagon-like peptide-1 analogues (GLP-1a) using administrative and healthcare registers.

**Methods:**

An active comparator design with a 3-year follow-up period was used. The main outcome was the hospital admission with a diagnosis of mild CI or major CI. Multivariate Cox Regression analysis was performed using the high-dimensional propensity score to obtain adjusted Hazard Ratio (HR) estimates. Inverse probability of treatment weighting (IPTW) and marginal structured model were used to calculate risk differences while accounting for the variations of confounders throughout the follow-up period.

**Results:**

Our results show a significant higher risk of major CI between DPP-4i and GLP-1a in unadjusted [HR (95% CI) = 3.13 (2.45–4.00), *p* < 0.001] and adjusted analyses [HR (95% CI) = 1.58 (1.22–2.06), *p* = 0.001]. No statistically significant differences were observed for mild CI. IPTW resulted stable throughout the follow-up period. Marginal structure modeling (β (95% CI) = 0.022 (0.020–0.024), *p* < 0.001) resulted in a higher risk of major CI for DPP-4i when compared to GLP-1a.

**Discussion:**

DPP-4i was associated with an increased risk of developing major CI when compared to GLP-1a among older individuals with type-2 diabetes.

**Supplementary Information:**

The online version contains supplementary material available at 10.1007/s00415-024-12300-9.

## Introduction

As the prevalence of type-2 diabetes continue to rise, there is a growing interest in understanding its impact on major and mild cognitive impairment (CI), especially among older individuals. CI poses a significant burden on older patients diagnosed with type-2 diabetes, substantially impacting their quality of life and overall health outcomes [1, 2]. Research suggests a complex interplay between diabetes-related factors, such as hyperglycaemia, insulin resistance, and chronic inflammation, contributing to cognitive decline. These impairments often manifest as deficits in memory, attention, processing speed, and executive function. Moreover, the presence of vascular complications exacerbates cognitive decline in diabetic patients. The multifaceted nature of this relationship underscores the urgent need for targeted interventions and comprehensive management strategies to alleviate the cognitive burden associated with type-2 diabetes, especially among older individuals, thereby enhancing the holistic well-being of affected individuals [1, 2].

Studies have shown that the prevalence of major CI in type-2 diabetes patients is approximately 15–25%, while the prevalence of mild CI is approximately 30–60%. This is not surprising considering that type-2 diabetes is a risk factor for both major and mild CI [2].

Recent literature emphasizes the necessity to optimize the pharmacological treatment in type-2 diabetes to effectively mitigate the risk of major and mild CI, particularly among individuals at an elevated risk of these clinical conditions. Specifically, there is a recognized need for head-to-head studies aiming to identify the optimal treatment for individuals with type-2 diabetes at an increased risk of major and mild CI [3].

Incretin-based treatments, which are widely used glucose-lowering drugs, have recently shown promise in improving cognitive functions among individuals with type-2 diabetes, positioning themselves as potential therapeutic agents for major CI [4, 5]. Notably, a recent meta-analysis of cohort studies revealed that dipeptidyl peptidase-4 inhibitors (DPP-4i) can significantly improve cognitive functions in patients with type-2 diabetes compared to other classes of glucose-lowering agents (i.e., sulfonylurea and metformin) [4]. In addition, glucagon-like peptide-1 analogues (GLP-1a) have garnered increased attention as pharmacological treatments capable of improving cognitive functions, supported by positive results from preclinical studies and some observational research [6, 7].

Furthermore, to the best of our knowledge, no head-to-head studies investigating the risks of major and mild CI have been conducted between DPP-4i and GLP-1a using real-world data.

This register-based study utilized the Danish Administrative and Healthcare Registers to compare the risk of a new diagnosis of major CI or mild CI among individuals aged 65 or older exposed to DPP-4i versus those exposed to GLP-1a.

## Methods

### Study design and data source

This registry-based cohort study utilized data from six Danish Administrative and Healthcare Registers, including the Danish Civil Registration System [8], the Danish National Patient Register [9], The Danish Register of Causes of Death [10], the Danish National Prescription Registry [11], the Population Education Registry [12], and the nationwide Register of Laboratory Results for Research [13]. Each Danish citizen, at birth or immigration, is assigned a personal civil registration (CPR) number, which serves as a unique identifier in national administrative databases. The CPR number enables access to comprehensive, virtually lifelong information on demographic characteristics, cause of death, claimed prescriptions at community pharmacies, hospital discharges, and laboratory measurements.

### Study population

**Fig. 1:**
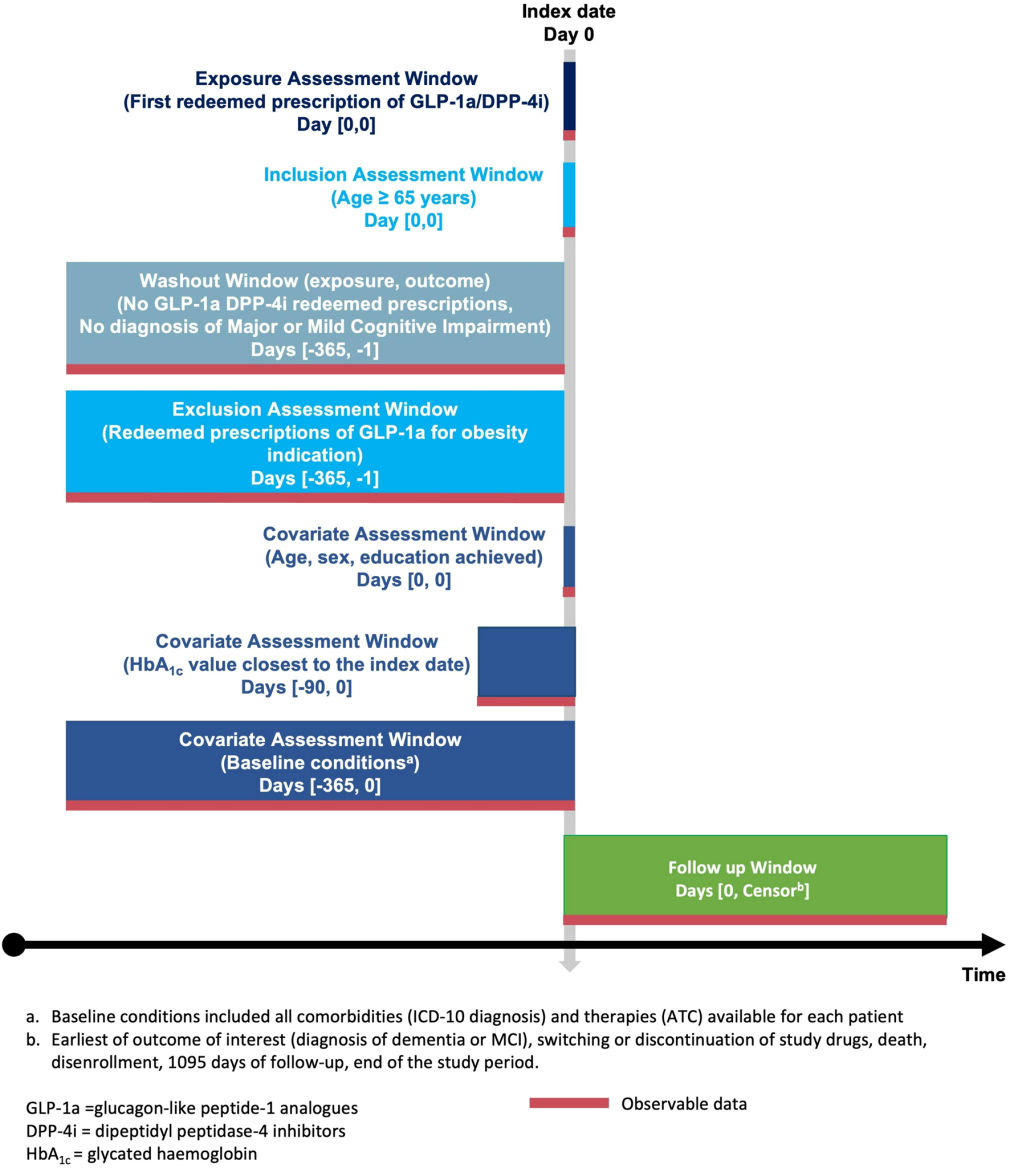
Study design diagram

The source population encompassed all Danish citizens aged 65 years and older with type-2 diabetes between 2007 and 2018 [11]. From this source population, our study population was selected, consisting exclusively of individuals with at least one redeemed prescription for GLP-1a [Anatomical Therapeutic Chemical (ATC) system: A10BJ] or DPP-4i [ATC code: A10BH] for type-2 diabetes. Specifically, GLP-1a included exenatide (A10BJ01) and liraglutide (A10BJ02), while DPP-4i included sitagliptin (A10BH01), vildagliptin (A10BH02), saxagliptin (A10BH03), alogliptin (A10BH04), and linagliptin (A10BH05). The study population was tracked through Danish registries from the date of the first redeemed prescription for the drugs of interest, known as the index date (Fig. 1).

The study population was divided into two cohorts: individuals treated with DPP-4i and those treated with GLP-1a.

### Follow-up

Individuals in the two cohorts were followed for 3 years from the index date until the occurrence of the outcome or were censored in case of emigration, the end of follow-up, death, or the end of data coverage. Censoring also applied to individuals who switched from one treatment to the other. The period 2007–2018 for registered prescriptions was chosen to ensure a minimum of 3 years of follow-up for all included subjects. The decision for a 3-year follow-up period was based on a previous Danish study, which reported a median follow-up time of 3.5 years before the occurrence of a major CI diagnosis in Randomized Controlled Trials [14]. However, in registers, a distinction between those with or without major CI treated with GLP-1a was observed already after 2–3 years of treatment [14].

### Outcomes

The main outcomes were hospital admission with a primary diagnosis of mild CI (ICD-10: G31.84, and F06.7) and hospital admission with a diagnosis of major CI, defined as a diagnosis of dementia in Alzheimer’s disease (ICD-10: F00), vascular dementia (ICD-10: F01), dementia in other diseases classified elsewhere (ICD-10: F02), unspecified dementia (ICD-10: F03), Alzheimer’s disease (ICD-10: G30), other degenerative diseases of the nervous system, not elsewhere classified (ICD-10: G31.0, G31.1, G31.83). Primary diagnoses were obtained from the Danish National Patient Register [9] and the mentioned ICD-10 codes were carefully checked used as guideline the Diagnostic and Statistical Manual of Mental Disorders, Fifth Edition, Text Revision (DSM-5-TR) for major and mild neurocognitive disorders [15] and the recommended codes by Allan et al. [16]. The validity of major CI diagnoses in the Danish National Patient Register has been previously assessed and it was found to have positive predictive values more than 80% [17, 18].

### Statistical analysis

A descriptive analysis was conducted for all the variables assessed at the index date, including age, sex, HbA_1c_, educational level, and comorbidities. HbA_1c_ measurements were retrieved using the Nomenclature for Properties and Units code 27,300 from the nationwide Register of Laboratory Results for Research [13]. Educational level and comorbidities were retrieved from the Population Education Registry [12] and the Danish National Patient Register [9], respectively. The methods and results were reported following the HARmonized Protocol Template to Enhance Reproducibility (HARPER) guidelines [19] (Supplementary Table S1). Continuous variables were presented as mean and SD, while categorical variables were expressed as frequencies and percentages. Crude Cox Regression survival analysis was conducted to assess the Hazard Ratio (HR) with the corresponding 95% confidence interval (CI) for major and mild CI in the DPP-4i group compared to the GLP-1a group (intention-to-treat analysis).

Multivariate Cox regression analysis was performed using the high-dimensional propensity score (HDPS) to obtain adjusted HR estimates. The HDPS was calculated using all available information regarding prescription drugs and clinical discharges for each patient, following the methodology described by Shakibfar et al. [20–22]. The HDPS has demonstrated superiority over traditional adjustment approaches in pharmacoepidemiologic studies in a Nordic setting [20]. The positivity assumption of the HDPS was checked using a Love plot.

In all analyses, a *p* value <0.05 was considered statistically significant. Data management was performed using SAS, version 9.4 (SAS Institute Inc., Cary, NC, USA), and analyses were conducted using R, version 3.5.0.

### Sensitivity analysis

To ensure statistically significant results in adjusted Cox regression, three sensitivity analyses were conducted:

1. The first sensitivity analysis aimed to address potential variations in the presence of confounders over the follow-up. The HDPS was recalculated annually during the follow-up, as outlined in paragraph 2.5. Boxplots illustrating the distribution of the HDPS over the follow-up years were employed to depict any changes. Subsequently, the inverse probability of treatment weighting (IPTW) [23] was computed and used in marginal structure models [24]. The computation of outcomes was determined as the risk difference for the outcomes.
2. The second sensitivity analysis was performed to account for misclassification bias of the outcomes. Specifically, we wanted to check if potential misclassifications in the registers regarding a diagnosis of major CI could affect our results. Correction of HR estimates and 95% CI for bias due to outcome misclassification was carried out according to the Brenner and Gefeller’s methodology [25]. The analysis was performed considering non-differential misclassification and a sensitivity of 71%, which has been previously been observed in validation studies of these diagnoses in the Danish National Patient Register [25].
3. The third sensitivity analysis involved including treatment discontinuation (measured with adherence) as an additional censoring criterion. Treatment discontinuation with DPP-4i and GLP-1a was assessed using adherence. Adherence was calculated through the medication possession ratio (MPR) [26]. The duration of each individual medical prescription was calculated by considering the defined daily doses (DDD) provided by the WHO ATC in 2023, the amount of DDD redeemed at each dispensing, and the recommended dosage based on therapeutic indications in Denmark provided by Medicin.dk [27]. A grace period of 90 days was permitted, and the grace period was also considered at the end of the treatment episode, following the approach described by Pazzagli et al. [28–30].

### Ethics

In Denmark, every patient record/information is pseudonymized before analysis, eliminating the need for informed consent or ethical approval in registry-based studies. The University of Copenhagen, where the analysis occurred, and Statistics Denmark (project number 707278) hold utilization data approval from the Regional Capital Area Data Protection Agency.

## Results

### Baseline characteristics of the study population

The study encompassed a population of 36,115 individuals aged over 65 with type-2 diabetes [mean (±SD) = 69.7(±7.6) years] who had initiated treatment with either GLP-1a (*n* = 11,745; 32.5%) or DPP-4i (*n* = 24,370; 67.5%). Liraglutide was the most prescribed GLP-1a (*n* = 10,718; 91.3%), while sitagliptin held the highest prescription rate among DPP-4i (*n* = 16,961; 69.6%). Individuals treated with DPP-4i were older compared to those receiving GLP-1a (71 vs. 67 years), with a similar percentage of males in the GLP-1a group as in the DPP-4i group. Highly prevalent comorbidities included hypercholesterolemia (77.7%) and hypertension (72.7%) in the overall population. Hypercholesterolemia, hypertension, major depression, ischemic heart disease, obesity, infection, and COPD were more frequently observed in patients treated with GLP-1a, while cerebrovascular disease and schizophrenia were more prevalent in those treated with DPP-4i. Mean (±SD) HbA_1c_ values were higher in individuals treated with GLP-1a compared to DPP-4i [67.9 (±16.5) mmol/mol vs. 62.1 (±16.1) mmol/mol]. Additional demographic and clinical characteristics of the patients included in the study are detailed in Table 1, and the analysed covariates are presented in Supplementary Figure S1.

**Table 1:** Descriptive analysis of GLP-1a and DPP-4i users

	GLP-1a (*n* = 11,745)	DPP-4i (*n* = 24,370)	Total (*n* = 36,115)
Mean age (± SD)	67.2 (± 5.8)	71.0 (± 8.1)	69.7 (± 7.6)
HbA_1c_ (mmol/mol)—mean (± SD)	67.9 (± 16.5)	62.1 (± 16.1)	63.9 (± 16.3)
HbA_1c_—latest measurement prior to the index date—median (days)	19	27	21
Sex, *n* (%)			
Males	6898 (58.7)	13,945 (57.2)	20,843 (57.7)
Females	4847 (41.3)	10,425 (42.8)	15,272 (42.3)
Drugs (ATC code), *n* (%)			
Exenatide (A10BJ01)	1027 (8.7)		1027 (2.8)
Liraglutide (A10BJ02)	10,718 (91.3)		10,718 (29.7)
Sitagliptin (A10BH01)		16,961 (69.6)	16,961 (47.0)
Vildagliptin (A10BH02)		2915 (12.0)	2915 (8.1)
Saxagliptin (A10BH03)		1536 (6.3)	1536 (4.3)
Alogliptin (A10BH04)		228 (0.9)	228 (0.6)
Linagliptin (A10BH05)		2730 (11.2)	2730 (7.6)
Educational level			
No education	47 (0.4)	91 (0.4)	138 (0.4)
Compulsory school and 10th grade	4,733 (41.4)	10,717 (45.7)	15,450 (44.3)
Vocational education and training and adult education	257 (2.2)	576 (2.5)	833 (2.4)
Upper secondary certificate (gymnasium)	282 (2.5)	658 (2.8)	940 (2.7)
Academic profession degrees	4187 (36.6)	8004 (34.1)	12,191 (34.9)
Bachelor and diploma degree	1075 (14.9)	2926(12.5)	4631(13.3)
Candidatus and master’s degree	159 (1.4)	332 (1.4)	491 (1.4)
PhD	19 (0.2)	30 (0.1)	49 (0.1)
Other	54 (0.5)	126 (0.5)	180 (0.5)
Comorbidities *n* (%)			
Hypertension	8982 (76.5)	17,276 (70.9)	26,258 (72.7)
Traumatic brain injury	354 (3.0)	753 (3.1)	1107 (3.1)
Schizophrenia	24 (0.2)	81 (0.3)	105 (0.3)
Major depression	1826 (15.5)	3478 (14.3)	5304 (14.7)
Bipolar disorders	28 (0.2)	51 (0.2)	79 (0.2)
Ischemic heart disease	3530 (30.1)	6518 (26.7)	10,048 (27.8)
Cerebrovascular disease	1274 (10.8)	3281 (13.5)	4555 (12.6)
Obesity	3555 (30.3)	6614 (14.8)	7169 (19.9)
Hypercholesterolemia	9624 (81.9)	18,447 (75.7)	28,071 (77.7)
Infection	5854 (49.8)	11,707 (48.0)	17,561 (48.6)
COPD	1980 (16.9)	3850 (15.8)	5830 (16.1)
Inflammatory disease	1559 (13.3)	3224 (13.2)	4783 (13.2)
Alcohol use disorder	377 (3.2)	857 (3.5)	1234(3.4)

*ATC* Anatomical Therapeutic Chemical system code, *COPD* chronic obstructive pulmonary disease, *DPP-4i* dipeptidyl peptidase-4 inhibitors, *GLP-1a* glucagon-like peptide-1 analogues

### Major and mild cognitive impairment

A total of 525 patients (1.5%) were registered with a diagnosis of major CI, while 79 patients (0.2%) were diagnosed with mild CI. Cox regression analysis revealed a significant difference for major CI between users of DPP-4i and GLP-1a in both crude [HR (95% CI) = 3.13 (2.45–4.00), *p* < 0.001] and adjusted analyses [HR (95% CI) = 1.58 (1.22–2.06), *p* = 0.001]. However, no statistically significant differences were observed for mild CI between GLP-1a and DPP-4i users in both crude [HR (95% CI) = 1.62 (0.97–2.71), *p* = 0.07] and adjusted analyses [HR (95% CI) = 1.32 (0.75–2.33), *p* = 0.34] (Table 2).

**Table 2:** Univariate and multivariate cox regression for major and mild cognitive impairment

Outcome	Drug classes	N. events (%)	Crude HR (95% CI)	Adjusted HR (95% CI)
Major cognitive impairment	GLP-1a (Ref. group)	81 (0.7)	–	–
	DPP-4i	444 (1.8)	3.13 (2.45–4.00)	1.58 (1.22–2.06)
Mild cognitive impairment	GLP-1a (Ref. group)	25 (0.2)	–	–
	DPP-4i	54 (0.2)	1.62 (0.97–2.71)	1.32 (0.75–2.33)

*HR* hazard ratio, *CI* confidence interval, *DPP-4i* dipeptidyl peptidase-4 inhibitors, *GLP-1a* glucagon-like peptide-1 analogues

### Sensitivity analysis

**Fig. 2:**
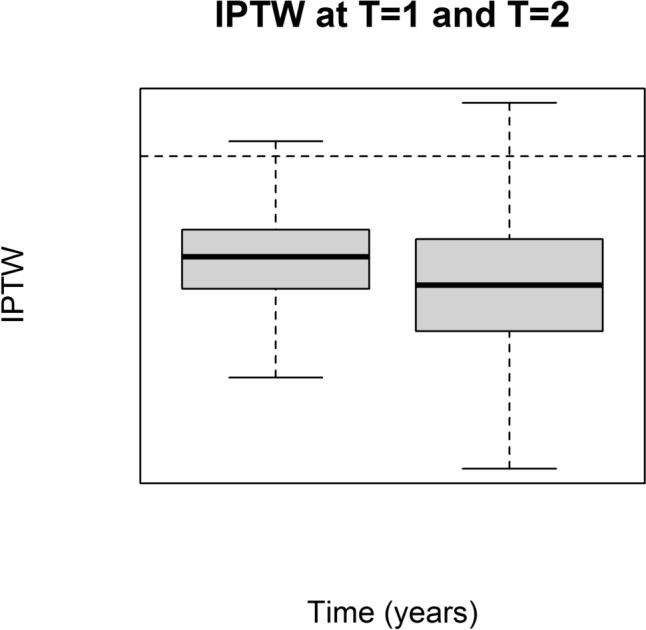
Inverse probability of treatment weighting (IPTW) after 1 and 2 years of follow-up

In the first sensitivity analysis, IPTW was calculated, and its stability was maintained throughout the follow-up period (Fig. 2). Marginal structure modeling revealed a positive association for DPP-4i (β (95% CI) = 0.022 (0.020–0.024), *p* < 0.001) with major CI, indicating a higher risk for major CI in the cohort exposed to DPP-4i compared to those exposed to GLP-1a.

In the second sensitivity analysis, we observed no discernible impact of misclassification of the study outcome on the results of the main analysis (Supplementary Figure S2).

In the third sensitivity analysis, adherence rates for both drugs were high with MPR (95% CI) at 102 (98–114)% for DPP-4i and 82 (73–106)% for GLP-1a, respectively. Even when treatment discontinuation was considered as an additional censoring criterion, Cox Regression analysis for major CI remained significant both before [HR (95% CI) = 8.4 (5.73–12.30), *p* < 0.001] and after adjustment [HR (95% CI) = 4.03 (2.71–5.98), *p* = 0.002], highlighting a major risk associated with DPP-4i compared to GLP-1a.

## Discussion

To the best of our knowledge, this is the first head-to-head study evaluating the impact of DPP-4i and GLP-1a on neurocognitive outcomes (i.e., major and mild CI) in older patients with type-2 diabetes, using real-world data. Our results show a statistically significant difference in major CI risk between DPP-4i and GLP-1a, both before and after adjustment, with notably higher risk associated with DPP-4i. Similar findings have been reported in existing scientific literature.

It should highlight that previous studies conducted in Denmark have observed a reduced risk of major CI among GLP-1a users when compared to non-users. Specifically, a nested case–control study encompassing all antidiabetic drugs in the Danish population showed a lower OR for major CI among individuals exposed to GLP-1a in comparison to those not exposed to pharmacological treatment for type-2 diabetes (OR: 0.58; 95% CI: 0.50–0.67) [31]. In another case–control study, each Danish patient exposed to GLP-1a exhibited the lowest risk of major CI compared to all other antidiabetic drugs combined (no head-to-head comparisons) [HR (95% CI) = 0.89 (0.86–0.93)] [14].

While we did not conduct mechanistic pharmacological studies, we speculated and hypothesized, drawing on evidence from pharmacological research, that the pharmacological action of DPP4-i and their inability to cross the blood–brain barrier offer a plausible explanation for differences in the risk of developing major and mild CI when compared to GLP-1a [6, 31, 32]. Specifically, GLP-1a can cross the blood–brain barrier, exhibiting pro-cognitive effects by improving neurogenesis, inflammation, oxidative stress, and cerebral glucose metabolism [31, 33, 34]. On the other hand, the DPP-4i linagliptin does not cross the blood–brain barrier and exhibits neuroprotective effects primarily attributed to peripheral functions through the inhibition of GLP-1 degradation [32]. Linagliptin has been proved to be a good substrate for P-glycoprotein, a factor that may significantly contribute to the drug’s efflux from the rat’s brain, despite its extensive distribution to other organs. The study also suggested that physiological alterations in Alzheimer’s disease could potentially result in reduced blood–brain barrier resistance, allowing for better penetration of the drug into the central nervous system [35, 36]. Another recent study found that omarigliptin, a novel once-weekly DPP-4i, can cross the blood–brain barrier due to its lipophilic properties [36]. The relationship between DPP-4i and cognitive function is complex and not fully understood. It may involve interactions with various factors such as age, disease duration, the presence of diabetic complications, and individual genetics. It is noteworthy that this protective effect of DPP-4i might be more pronounced in younger patients and those without diabetic complications [37]. In addition, we observed that individuals who had a redeemed prescription for GLP-1a had mean higher baseline HbA_1c_ values measured closest to the index date, potentially underestimating the risk of major CI for DPP4-i, rather than overestimating it. This aligns with the findings of a previous study that showed an increased risk of major CI in type-2 diabetes patients with rising HbA_1c_ (a proxy for poor glycaemic control) compared to those with lower levels [38].

IPTW consistently indicated a positive association between DPP-4i and major CI, reinforcing our primary findings. The robustness of our results was further supported by high adherence levels in both drug classes, as evidenced by MPR. Notably, patients with a redeemed prescription of DPP-4i exhibited better adherence, suggesting that differences in outcomes were not attributed to variations in medication compliance. Furthermore, even after including treatment discontinuation as an additional censoring criterion during the follow-up, Cox regression analysis continued to reveal a significant and substantial risk of major CI associated with DPP-4i exposure.

### Strengths and limitations

The study’s strengths lie in its population-based approach and the extensive analysis of a large number of subjects encompassing the entire Danish population [39]. Furthermore, the use of rigorous statistical methods effectively minimized residual confounding [23, 24]. However, the study has its limitations. The number of patients experiencing the outcomes was relatively low, possibly due to potential misclassification of major and mild CI, especially for non-severe cases that did not require hospitalization. In cases where the disease is mild and managed in outpatient settings by healthcare professionals, such diagnoses may not be recorded in the National Patient Register. In addition, the available data only included diagnoses based on clinical admissions for major and mild CI, often established in outpatient clinics. It is essential to acknowledge that while the validation study conducted by Phung et al. [17] demonstrated higher validity in diagnosing major CI, it may not fully capture the complexities inherent in real-world diagnostic scenarios and their associated validity.

## Conclusion

The use of DPP-4i was associated with a higher risk of developing major CI in comparison to GLP-1a users among older individuals with type-2 diabetes during the initial 3 years of treatment in Denmark from 2007 to 2018. Future research is necessary to validate these results and delve into the underlying mechanisms. In addition, it should more thoroughly evaluate the clinical relevance of these findings for older patients with type-2 diabetes.

### Supplementary Information

Below is the link to the electronic supplementary material.

Supplementary file1 (DOCX 34 KB)

Supplementary file2 (DOCX 337 KB)

## Data Availability

Data are stored on secure servers on Statistics Denmark and cannot be shared according to Statistics Denmark regulations. Access to Statistics Denmark servers and the associated data can be granted by Statistics Denmark upon adequate permissions.
