# Supplementary material for: Comparing major and mild cognitive impairment risks in older type-2 diabetic patients: a Danish register-based study on dipeptidyl peptidase-4 inhibitors vs. glucagon-like peptide-1 analogues
Source: J Neurol. 2024 Mar 22;271(6):3417–25. doi: 10.1007/s00415-024-12300-9 (PMC11136777; doi:10.1007/s00415-024-12300-9)
Supplement: Supplementary file 2 — Supplementary file2 (DOCX 337 KB) [file 415_2024_12300_MOESM2_ESM.docx]

**Supplementary Material**


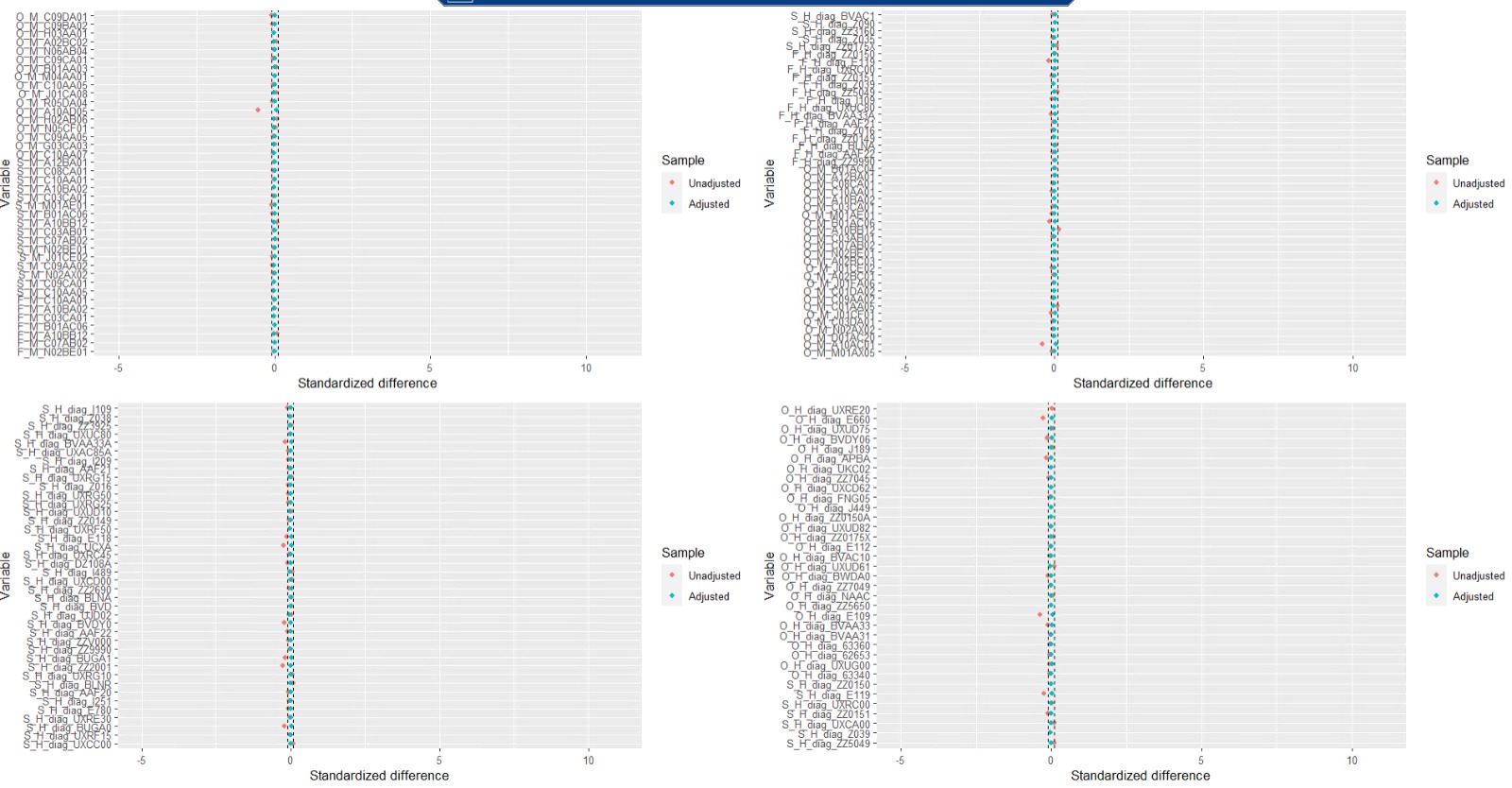


**Fig. S1.** Check for positive assumption of covariates before and after the adjustment.

Red dots are unadjusted values for standardized mean difference between the groups, while blue dotes represent the difference after the adjustment.

**
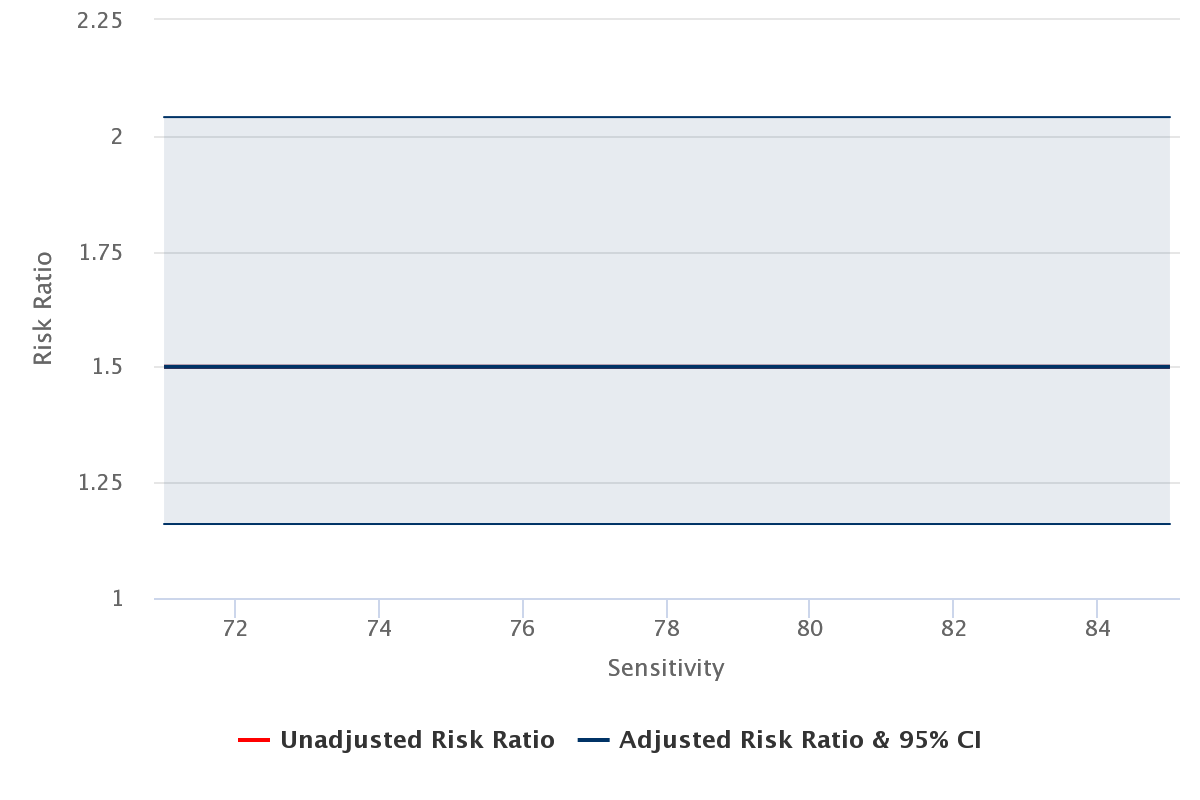
**

**Fig. S2.** Sensitivity analysis for misclassification bias on the diagnosis of dementia in older patients treated with dipeptidyl peptidase-4 inhibitors (DPP4-i) or glucagon-like peptide-1 analogues (GLP-1a).

**Table S1.** Full list of all inclusion and exclusion criteria (a), exposure (b), outcome (c), patient’s characteristics (d), and registers (e) used in the study in accordance with the HARmonized Protocol Template to Enhance Reproducibility (HARPER) guidelines.

a)

| **Inclusion criteria** | **Order of application** | **Assessment window** | **Code Type** | **Applied to study populations** |
| --- | --- | --- | --- | --- |
| Age ≥ 65 years | Before selection of index date | [0,0] | n/a | Exposure: DPP4-i  Comparator: GLP-1a |
| **Exclusion criteria** | **Order of application** | **Assessment window** | **Code Type** | **Applied to study populations** |
| Dispensing of GLP-1a | Before selection of index date | [-365, -1] | ATC | Exposure: DPP4-i  Comparator: GLP-1a |
| Dispensing of DPP-4i | Before selection of index date | [-365, -1] | ATC | Exposure: DPP4-i  Comparator: GLP-1a |
| Dispensing of drugs with obesity indication | Before selection of index date | [-365, -1] | Brand name:  Wegowy^®^, semaglutide; Saxenda^®^, liraglutide | Exposure: DPP4-i  Comparator: GLP-1a |
| Dementia diagnosis | Before selection of index date | [-365, -1] | ICD-10 | Exposure: DPP4-i  Comparator: GLP-1a |

b)

| **Exposure group name** | DPP-4i | GLP-1a |
| --- | --- | --- |
| **Details** | Oral formulation | Injection formulation |
| **Washout window** | [-365, -1] | [-365, -1] |
| **Assessment window** | [1, censor] | [1, censor] |
| **Code Type** | ATC | ATC |
| **Applied to study populations** | Exposure | Comparator |
| **Incident with respect to** | Incident user of DPP-4i, exclude the patients with GLP-1a at the same time at the start point | Incident user of GLP-1a, exclude the patients with DPP-4i at the same time at the start point |
| **Measurement characteristics/ validation** | Coverage: >99% [1] | Coverage: >99% [1] |

c)

| **Outcome definition** | **Code Type** | **Applied to study populations** | **Measurement characteristics/**  **validation** | **Assessment period** | **Source of algorithm** |
| --- | --- | --- | --- | --- | --- |
| Diagnosis of major cognitive impairment | Dementia in Alzheimer’s disease (ICD-10: F00), vascular dementia (ICD-10: F01), dementia in other diseases classified elsewhere (ICD-10: F02), unspecified dementia (ICD-10: F03), Alzheimer’s disease (ICD-10: G30), other degenerative diseases of the nervous system, not elsewhere classified (ICD-10: G31.0, G31.1, G31.83) | Exposure: DPP4-i  Comparator: GLP-1a | Positive predicted values were more than 80% with the lowest validity in younger populations and for vascular dementia and with a sensitivity of 71% for dementia/vascular dementia/fronto-temporal dementia | [0, censor] | [2,3] |
| Diagnosis of mild cognitive impairment | G31.84, F06.7 | Exposure: DPP4-i  Comparator: GLP-1a |  | [0, censor] | [2,3] |

d)

| **Characteristic** | **Details** | **Type of variable** | **Assessment window** | **Code Type** | **Applied to study populations** | **Source of algorithm** |
| --- | --- | --- | --- | --- | --- | --- |
| Gender | Male/Female | Binary | [0,0] | n/a | Exposure: DPP4-i  Comparator: GLP-1a | n/a |
| Age | Age in years defined by (time 0 – _year of birth)/365 | Continuous | [0,0] | n/a | Exposure: DPP4-i  Comparator: GLP-1a | n/a |
| Comorbidity | Related to T2DM | Categorical | [-365, 0] | ICD-10 | Exposure: DPP4-i  Comparator: GLP-1a | [4-8] |
| Education achieved | No education  Compulsory school and 10th grade  Vocational education and training and adult education  Upper secondary certificate (gymnasium)  Academic profession degrees  Bachelor and diploma degree  Candidatus and master’s degree  PhD  Other  Missing | Categorical | [0,0] | n/a | Exposure: DPP4-i  Comparator: GLP-1a | [9] |
| HbA_1c_ levels | Laboratory levels of HbA_1c_ | Continuous | Restricted to the nearest value to the index date | NPU 27300 (mmol/mol  [IFCC]) | Exposure: DPP4-i  Comparator: GLP-1a | [10] |

e)

| **Register** | **Variables from this register** | **Coverage from year** | **Source of algorithm** |
| --- | --- | --- | --- |
| Civil Personal Registry | CPR number  sex  date of birth  vital status (death) | 1968 (Greenland Region 1972) | [11] |
| National Patient Registry | diagnosis of type 2 diabetes  diagnosis of dementia  comorbidities | 1977 | [12] |
| Danish National Prescription Registry | DPP4-i prescriptions  GLP-1a prescriptions | 1994 | [13] |
| National Laboratory Database | HbA_1c_ measurement | 2008 | [14] |
| Population Education Registry | Education level | 1981 | [15] |

ATC, Anatomical Therapeutic Chemical system code; CPR, personal civil registration number; DPP-4i, dipeptidyl peptidase-4 inhibitors; GLP-1a, glucagon-like peptide-1 analogues; NPU, Nomenclature for Properties and Units.

**References**

1. Sundhedsdatastyrelsen - Statistikker [Internet]. [cited 2023 Nov 14]. Available from: <https://medstat.dk/>
2. Phung TKT, Andersen BB, Høgh P, Kessing LV, Mortensen PB, Waldemar G. Validity of dementia diagnoses in the Danish hospital registers. Dement Geriatr Cogn Disord. 2007;24:220–8.
3. Thygesen SK, Christiansen CF, Christensen S, Lash TL, Sørensen HT. The predictive value of ICD-10 diagnostic coding used to assess Charlson comorbidity index conditions in the population-based Danish National Registry of Patients. BMC Med Res Methodol. 2011;11:83.
4. Wium-Andersen IK, Osler M, Jørgensen MB, Rungby J, Wium-Andersen MK. Antidiabetic medication and risk of dementia in patients with type 2 diabetes: a nested case–control study. Eur J Endocrinol. 2019;181:499–507.
5. Mateu NC. Traumatic brain injury in Denmark 2008-2012. Scand J Public Health. 2020;48:331–7.
6. Sessa M, Mascolo A, Andersen MP, Rosano G, Rossi F, Capuano A, et al. Effect of Chronic Kidney Diseases on Mortality among Digoxin Users Treated for Non-Valvular Atrial Fibrillation: A Nationwide Register-Based Retrospective Cohort Study. PloS One. 2016;11:e0160337.
7. Köhler-Forsberg O, Antonsen S, Pedersen CB, Mortensen PB, McGrath JJ, Mors O. Schizophrenia spectrum disorders in Denmark between 2000 and 2018: Incidence and early diagnostic transition. Acta Psychiatr Scand. 2023;148:190–8.
8. Jefsen OH, Erlangsen A, Nordentoft M, Hjorthøj C. Cannabis Use Disorder and Subsequent Risk of Psychotic and Nonpsychotic Unipolar Depression and Bipolar Disorder. JAMA Psychiatry. 2023;80:803–10.
9. Hvidberg MF, Frølich A, Lundstrøm SL. Catalogue of socioeconomic disparities and characteristics of 199+ chronic conditions-A nationwide register-based population study. PloS One. 2022;17:e0278380.
10. Labterm Webportal [Internet]. [cited 2023 Nov 14]. Available from: <https://www.labterm.dk/webportal/frontpage>
11. Pedersen CB. The Danish Civil Registration System. Scand J Public Health. 2011;39:22–5.
12. Schmidt M, Schmidt SAJ, Sandegaard JL, Ehrenstein V, Pedersen L, Sørensen HT. The Danish National Patient Registry: a review of content, data quality, and research potential. Clin Epidemiol. 2015;7:449–90.
13. Kildemoes HW, Sørensen HT, Hallas J. The Danish National Prescription Registry. Scand J Public Health. 2011;39:38–41.
14. Arendt JFH, Hansen AT, Ladefoged SA, Sørensen HT, Pedersen L, Adelborg K. Existing Data Sources in Clinical Epidemiology: Laboratory Information System Databases in Denmark. Clin Epidemiol. 2020;12:469–75.
15. Jensen VM, Rasmussen AW. Danish education registers. Scand J Public Health. 2011;39:91–4.
